# Risk factors for parastomal hernia in Japanese patients with permanent colostomy

**DOI:** 10.1007/s00595-013-0721-3

**Published:** 2013-09-13

**Authors:** Kimihiko Funahashi, Takayuki Suzuki, Yasuo Nagashima, Satoshi Matsuda, Junichi Koike, Hiroyuki Shiokawa, Mitsunori Ushigome, Kenichiro Arai, Tomoaki Kaneko, Akiharu Kurihara, Hironori Kaneko

**Affiliations:** Department of General and Gastroenterological Surgery, Toho University Medical Center Omori Hospital, 6-11-1 Omorinishi Otaku, Tokyo, 143-8541 Japan

**Keywords:** Parastomal hernia, Risk factor, Colostomy

## Abstract

**Purpose:**

Although the definitive risk factors for parastomal hernia development remain unclear, potential contributing factors have been reported from Western countries. The aim of this study was to identify the risk factors for parastomal hernia in Japanese patients with permanent colostomies.

**Methods:**

All patients who received abdominoperineal resection or total pelvic exenteration at our institution between December 2004 and December 2011 were reviewed. Patient-related, operation-related and postoperative variables were evaluated, in both univariate and multivariate analyses, to identify the risk factors for parastomal hernia formation.

**Results:**

Of the 80 patients who underwent colostomy, 22 (27.5 %) developed a parastomal hernia during a median follow-up period of 953 days (range 15–2792 days). Hernia development was significantly associated with increasing patient age and body mass index, a laparoscopic surgical approach and the transperitoneal route of colostomy formation. In the multivariate analysis, the body mass index (*p* = 0.022), the laparoscopic approach (*p* = 0.043) and transperitoneal stoma creation (*p* = 0.021) retained statistical significance.

**Conclusions:**

Our findings in Japanese ostomates match those from Western countries: a higher body mass index, the use of a laparoscopic approach and a transperitoneal colostomy are significant independent risk factors for parastomal hernia formation. The precise role of the stoma creation route remains unclear.

## Introduction

Advances in surgical techniques have enabled more frequent sphincter preservation in patients with rectal malignancy, but have not eliminated the need for permanent colostomy. Although the majority of ostomates (patients with stomata) seem to adapt well after a period of time, those with stoma-related complications face many problems. A parastomal hernia, defined as an incisional hernia at the site of an intestinal stoma, is a late complication with an incidence of 0–48 % in ostomates with loop- or end colostomies, according to a review by Carne et al. [1]. Although obesity, corticosteroid use, increasing age and wound infection are believed to be potential contributing factors to parastomal hernia in Western countries [2], the definitive risk factors are still unclear; this is particularly true in Eastern countries, including Japan. In 2012, Korean researchers found that female sex, aperture size, age over 60 years, a body mass index (BMI) >25 kg/m^2^ and hypertension are probable risk factors for parastomal hernia [3, 4], but there is still controversy over defining these risk factors, and there have been no reports specifically addressing Japanese patients. As the laparoscopic approach to abdominoperineal resection (APR) is gaining widespread acceptance among surgeons [5–9], the risk factors for parastomal hernia development must be verified. We conducted a retrospective study to identify independent risk factors for parastomal hernia formation in Japanese patients with permanent colostomies, and evaluated the differences in the hernia rates between Japanese and Western ostomates.

## Methods

Between December 2004 and December 2011, APR or total pelvic exenteration (TPE) was performed in 80 patients at the Toho University Medical Center Omori Hospital in Tokyo, Japan. After patients were discharged from the hospital, we routinely performed abdominal computed tomography (CT) and obtained serum tumor markers, such as carcinoembryonic antigen and carbohydrate 19-9, every 3–6 months to monitor patients for recurrence or metastasis. When patients visited the hospital for outpatient follow-up, we conducted examinations in both the supine and standing positions to look for parastomal hernias. When a hernia was suspected, we performed abdominal CT to confirm the finding.

A univariate and multivariate analysis of 12 clinical variables was conducted, comparing the parastomal hernia group with the control group. The presence or absence of a parastomal hernia was used as the dependent variable, and the clinical characteristics evaluated were the independent variables: sex, age, BMI, comorbidities [diabetes mellitus (DM) and chronic obstructive pulmonary disease (COPD)], elective or emergency operation, the type of procedure (APR or TPE), approach (open or laparoscopic), route of stoma creation (extraperitoneal or transperitoneal), the role of the attending surgeon (primary surgeon or assistant), past history of open laparotomy, wound infection and presence of an incisional hernia at the median wound. Extraperitoneal colostomy was performed according to Goligher’s technique [10]. Transperitoneal colostomies were created using a round incision at the preoperatively marked skin site. The anterior rectal sheath was incised in a cruciate fashion. The rectus abdominis muscle was split to expose the posterior rectal sheath; both this structure and the peritoneum were cut longitudinally. Allis forceps were then used to grasp the stump of the colon and to pull it through the skin incision. Finally, the colon was fixed to both rectal sheaths at four points using monofilament absorbable 4-0 PDS-II^®^ sutures (Ethicon Endo-Surgery, Inc., San Angelo, TX, USA).

### Statistical analyses

Patients with parastomal hernias were compared with controls (no hernia) using *χ* ^2^ and Mann–Whitney *U* testing for categorical and continuous data, respectively. All factors that were significant in the univariate analysis were entered into a multivariate stepwise logistic regression with backward elimination to identify the independent factors. All data were entered in a computer database and analyzed using the Statistical Package for the Social Sciences (SPSS) for Windows software program, version 9.0.2 (SAS Institute Inc., Cary, NC, USA). Differences were considered significant for values of *p* < 0.05.

## Results

### Patient characteristics

The patient characteristics are shown in Table 1. There were 80 total patients, 59 males and 21 females, with a median age of 66 years (range 33–90 years). The median BMI was 21.4 kg/m^2^ (range 15.0–32.8 kg/m^2^). DM was present in 32 patients (40.0 %), and 20 patients (25.0 %) had COPD.

**Table 1:** Patient characteristics

	*n*
Sex	
Male	59
Female	21
Median age (range) (years)	66 (33–90)
Median BMI (range) (kg/m^2^)	21.4 (15.0–32.8)
Comorbidity	
DM	32
COPD	20
Laparotomy during the follow-up period	25
Disease	
Rectal cancer	55
Anal cancer	8
Local recurrence of malignancy	10
Malignant melanoma	2
Rectal metastasis (gastric cancer)	1
Prostate cancer	1
Ovarian cancer	1
Ischemic colitis	2
Procedure	
APR	67
TPE	13
Elective/emergency	
Elective	78
Emergency	2
Type of approach	
Laparoscopic	8
Open	72
Parastomal hernia	
No	58
Yes	22
Median follow-up period (range) (days)	953 (15–2792)

*BMI* body mass index, *DM* diabetes mellitus, *COPD* chronic obstructive pulmonary disease, *APR* abdominoperineal resection, *TPE* total pelvic exenteration

Of the 80 total patients who underwent amputation of the rectum, 76 had anorectal malignancy, two had ischemic colitis, one had prostatic cancer and one had ovarian cancer. APR was performed in 67 patients (84.0 %) and TPE in 13 patients. Only the two patients with ischemic colitis underwent emergency surgery. The laparoscopic approach was used in only eight of the 67 patients (11.9 %) who underwent APR for anorectal cancer; in six of these eight patients, the colostomy was performed transperitoneally. The first author (KF) took part in 50 of the 80 procedures as the primary surgeon. Wound infections developed in eight patients (10.0 %). There were 25 patients (31.3 %) who underwent laparotomy during a median follow-up period of 953 days (range 15–2792 days). An incisional hernia at the median wound was identified in seven patients (8.8 %) by routine abdominal CT.

Parastomal hernias were identified in 22 (27.5 %) of the 80 ostomates. The control group was made up of the remaining 58 patients. The median period from stoma creation until the identification of a parastomal hernia was 302 days (range 11–1829 days). Only one patient required repair with expanding polytetrafluoroethylene mesh, because of bowel incarceration.

### Results of the univariate and multivariate analyses

The incidence of parastomal hernia formation was significantly associated with increasing patient age and BMI, the laparoscopic approach and the transperitoneal route of stoma creation (Table 2). An increasing BMI, the laparoscopic approach and the transperitoneal route of stoma creation retained statistical significance in the multivariate analysis (Table 3).

**Table 2:** Risk factors for parastomal hernia formation (univariate analysis)

Variable	No. of patients		Univariate
	Parastomal hernia (+)	Parastomal hernia (−)	*p*
Sex			
Male	16	43	0.898
Female	6	15	
Age (years)	70.227 ± 2.403	63.000 ± 1.480	<0.05
BMI (kg/m^2^)	23.500 ± 0.693	21.164 ± 0.427	<0.01
DM			
Yes	12	20	0.139
No	10	38	
COPD			
Yes	7	13	0.393
No	15	45	
Past history of open laparotomy			
No	15	40	0.946
Yes	7	18	
Procedure			
APR	17	50	0.346
TPE	5	8	
Elective/emergency			
Elective	22	56	0.253
Emergency	0	2	
Type of approach			
Laparoscopic	6	2	<0.01
Open	16	56	
Route of stoma creation			
Transperitoneal	16	18	<0.01
Extraperitoneal	6	40	
Attending surgeon’s role			
Primary surgeon	14	36	0.897
Assistant	8	22	
Wound infection			
Yes	2	6	0.866
No	20	52	
Incisional hernia at main wound scar			
Yes	2	5	0.947
No	20	53	

*BMI* body mass index, *DM* diabetes mellitus, *COPD* chronic obstructive pulmonary disease, *APR* abdominoperineal resection, *TPE* total pelvic exenteration

**Table 3:** Independent significant factors predicting parastomal hernia formation (multivariate analysis)

Variable	*p*	95 % confidence interval	Odds ratio
Age	0.114	0.574–364.702	11.634
BMI (kg/m^2^)	0.022	1.698–1916.885	45.608
Type of approach (laparoscopic/open)	0.043	1.061–66.283	7.213
Route of stoma creation (transperitoneal/extraperitoneal)	0.021	1.226–13.975	3.964

*BMI* body mass index

## Discussion

Laparoscopic surgery for colon cancer has been performed in Japan since 1993, and this approach is rapidly increasing in popularity. More recently, laparoscopic APR for select rectal malignancies has been gaining acceptance among surgeons in Western countries [5–9]. It is well known that most ostomates face sensitive physical, social and psychological problems, and severe stoma-related issues often impair their quality of life. A parastomal hernia, known to be a common complication, often causes stoma-care problems, such as leakage and skin irritation, and can lead to rare but severe complications including obstruction, bowel incarceration and perforation. Although three of our patients had symptomatic parastomal hernias, only the patient with bowel incarceration underwent surgery. The other two did not undergo repair because of advanced age (87 years) and tumor progression, respectively. Nineteen of the 22 patients with parastomal hernias were managed conservatively. Parastomal hernia repair (primary suturing and stoma relocation) is required in ostomates facing severe complications or with poor adaptation to ostomy appliances, but the results of repair are generally disappointing [10–12]. Good results for parastomal hernia prevention using mesh have been reported since the 1990s, but adverse events such as infection, mesh erosion and the requirement of mesh removal have been reported [13–15]. Hernia prevention without the use of mesh is, therefore, the best management strategy, and surgeons should be familiar with the risk factors for parastomal hernias to help prevent their development.

Obesity, chronic lung disease, type II DM, advancing age, malnutrition, renal failure, malignancy, steroid treatment, jaundice, radiotherapy, chemotherapy and oral anticoagulant use are considered to be patient-related factors that increase the risk of developing an incisional hernia [16]. In addition to these factors, the site of stoma placement (i.e., through the abdominal rectus muscle), the peritoneal route used for colostomy creation (extraperitoneal or transperitoneal) and the size of the fascial opening are reported to be risk factors for parastomal hernia development [4, 17]. However, it is doubtful whether these parastomal hernia risk factors, identified in non-Japanese patients, apply to Japanese ostomates with permanent colostomies because of their environmental and genetic differences. In the present study, the site of stoma placement was excluded from the variable analysis, because abdominal CT performed during the follow-up showed that all stomas were created through the abdominal rectus muscle. The 11 remaining clinical variables were subjected to the analysis comparing the parastomal hernia group with the control group. A parastomal hernia was identified in 27.5 % of the 80 patients with permanent colostomy, half developing within 6 months of colostomy formation. Hernia development was significantly associated, in the univariate analysis, with increasing age and BMI, laparoscopic surgery and the transperitoneal route of stoma creation. The multivariate analysis revealed a statistically significant association between parastomal hernia formation and an increasing BMI, the laparoscopic approach and the transperitoneal route of stoma creation.

Obesity impacted the development of both incisional and parastomal hernias, and its importance has been emphasized by many previous researchers. Cobb et al. [18] reported that the BMI was significantly associated with the incidence of incisional hernia formation. Kouba et al. [2] evaluated stomal complications in 137 patients undergoing cystectomy with ileal conduit urinary diversion for bladder cancer, and reported that patients in whom complications develop have a significantly higher mean BMI than those without complications. Raet et al. [19] proposed placing prophylactic mesh during colostomy formation when the patient’s waist circumference exceeds 100 cm.

Since Goligher first described an extraperitoneal approach to colostomy in 1958 [20], this has become the traditional method. Surgeons, including those in our group, believe that extraperitoneal colostomy is superior to the transperitoneal route. The extraperitoneal approach reportedly has a significantly lower risk of herniation than the transperitoneal route in ostomates with permanent stomata. Lian et al. [21] conducted a meta-analysis of 1071 patients and reported that extraperitoneal colostomy is associated with a lower rate of postoperative parastomal hernia than transperitoneal colostomy, but pointed out that all studies included in the meta-analysis were retrospective in nature, and the number of patients undergoing extraperitoneal colostomy was small. In the present study, parastomal hernias developed more frequently in the laparoscopic group than in the open group. However, it is important to note that colostomy was performed transperitoneally in five of the six laparoscopic patients with parastomal hernias. Therefore, according to our very limited series, the route of stoma creation may be associated with the formation of a parastomal hernia.

Hamada et al. [22, 23] reported the usefulness of and the proper technique for extraperitoneal colostomy in laparoscopic APR, although the risk factors for hernia formation were not described. Extraperitoneal colostomy may be recommended for patients undergoing laparoscopic APR. Leroy et al. [24] reported, in a limited series, that the extraperitoneal colostomy approach in laparoscopic APR may reduce the risk of parastomal hernia formation.

In conclusion, we found that an increasing BMI, the laparoscopic surgical approach and the transperitoneal route of stoma creation are independent risk factors for parastomal hernia development. Our study in Japanese ostomates supports the data reported from Western countries. The transperitoneal route of stoma creation is considered to be a particularly important, independent risk factor for parastomal hernia development. However, the exact role of the stoma creation route in hernia formation is still unclear; a multicenter, randomized, controlled trial is needed to verify and further explain this finding.
